# Open fan sign: An ultrasound finding suggesting postpartum intrauterine forgotten gauze

**DOI:** 10.1002/ccr3.1886

**Published:** 2018-10-22

**Authors:** Liangcheng Wang, Tomoyuki Kuwata, Isao Horiuchi, Haruko Ariga, Ken Imai, Hiroyoshi Ko, Azusa Kimura, Kenro Chikazawa, Shiho Oide, Kenjiro Takagi

**Affiliations:** ^1^ Perinatal and Maternal Center of Saitama Medical Center Jichi Medical University Saitama Japan

**Keywords:** acoustic impedance, intrauterine forgotten gauze, open fan sign

## Abstract

Gauze counting is regarded as the most essential way to prevent forgotten gauze inside the body during any surgery. However, incident may still occur due to artificial mistake. An open fan sign on ultrasonography may indicate a gauze left in the intrauterine cavity.

## 1 DESCRIPTION

A 31‐year‐old woman had vaginal delivery complicated with grade III perineal tear 4 weeks ago. Several gauzes were temporarily inserted into the cervix and vagina for hemostasis during the repair procedure. On postpartum day 4, vaginal ultrasonography was performed, which was a routine procedure in this institute: At that time, we did not recognize any abnormalities. The patient was discharged on postpartum day 5.

**Figure 1:**
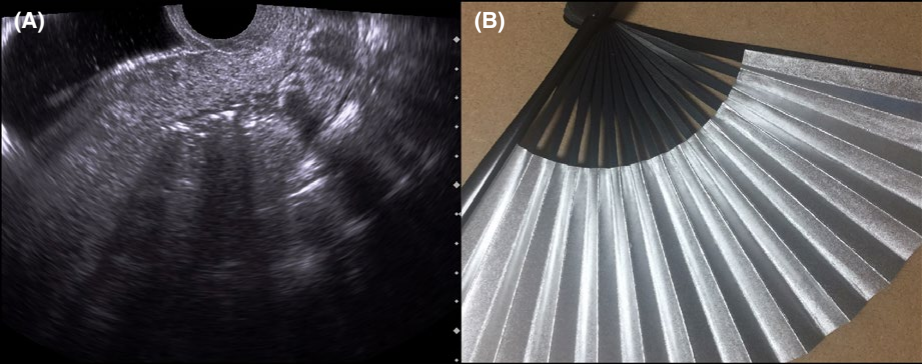
A, Ultrasonography on postpartum day 4. Acoustic shadows originating from the low intrauterine cavity were observed. B, An open fan

**Figure 2:**
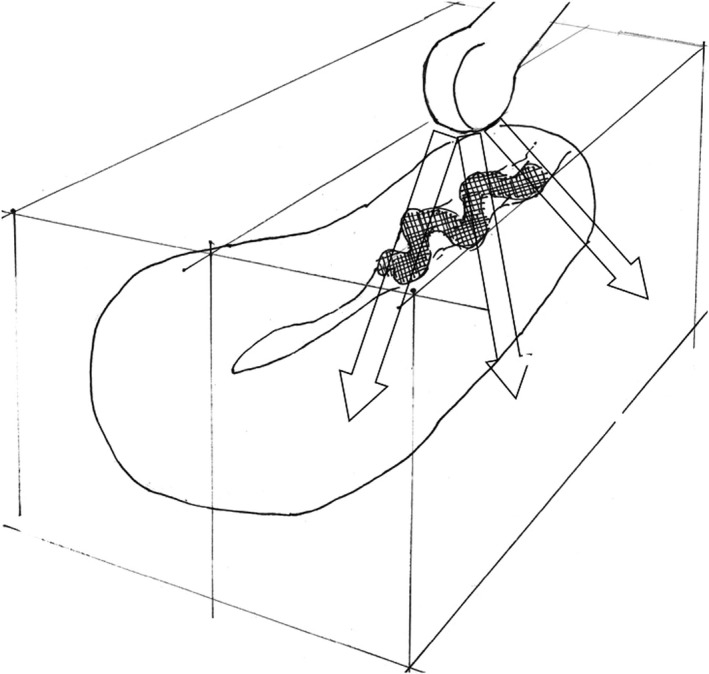
A gauze with a snake shape located on the lower intrauterine cavity toward the cervix

One month later, she visited us for a routine postpartum examination. Vaginal examination revealed an odorous smelling, brown‐colored gauze in the vagina, which was pulled out. Although not confirmed, the gauze was localized in the vagina, with no parts being located within the cervical canal. Ultrasonography on postpartum day 4 was reviewed once again. Acoustic shadow in the low intrauterine cavity was found (Figure 1A), mimicking an “open fan” (Figure 1B). An attenuation of ultrasound wave occurs when various substances with higher impedance difference exist in the interface.^1^ If this substance is located noncontinuously or has various thickness in the longitudinal scan direction, it may create an “open fan” like feature. We believe that a gauze, being located in the lower intrauterine cavity to cervix, thus making a snake‐like shape, caused this “open fan” sign (Figure 2). The gauzed dropped to the vagina, which was found at the regular checkup at one month postpartum. Although gauze counting is regarded as the most essential way to prevent a forgotten gauze inside the body during surgery, incident may still occur. An “open fan sign” on ultrasonography may suggest intrauterine (forgotten) gauze in postpartum period and, thus, may become addition to prevent it. This ultrasound sign may be applicable to detect a forgotten gauze in any organ other than the uterus/cervix depending on the gauze location and its surrounding structures although we have not yet data for it.

## CONFLICT OF INTEREST

No conflict of interests.

## AUTHOR CONTRIBUTIONS

LW and TK: conceived of the presented idea. LW, KC, and SO: involved in manuscript writing. KI: involved in postdelivery vagina repair procedure. IH, HA, HK, and AK: involved in the postpartum management of the patient. KT: supervised the findings of this work. All authors discussed the results and contributed to the final manuscript.
